# Biocompatibility of Human Fibroblast Cells Exposed to Gold Nanoparticles Synthesized by Environmentally Friendly Route

**DOI:** 10.1021/acsomega.6c06962

**Published:** 2026-09-30

**Authors:** Wellington Vieira de Souza, Keilla Gomes Machado, Francine Girardello, Amanda Poletto Santi, Noemi Raquel Checca Huaman, Daniel Ramos Louzada, Rubem L. Sommer, Fernando Joel Scariott, Rônei de Almeida Aragão, Cesar Aguzzoli, Mariana Roesch-Ely

**Affiliations:** † Posgraduate Program in Biotechnology, 58802 University of Caxias do Sul, Caxias do Sul, Rio Grande do Sul 95070560, Brazil; ‡ 74350 Brazilian Center for Research in Physics (CBPF), Rio de Janeiro, Rio de Janeiro 22290-180, Brazil; § Posgraduate Program in Materials Science and Engineering, 58802 University of Caxias do Sul, Caxias do Sul, Rio Grande do Sul 95070560, Brazil

## Abstract

Green toxicology emphasizes the need for safe, efficient, and environmentally clean processes for the production and manipulation of AuNPs. Given the significant concerns regarding health and environmental hazards, the biocompatibility of AuNPs must be thoroughly investigated at both cellular and molecular levels. Here, gold nanoparticles were synthesized by magnetron sputtering on a solid powder substrate through a new and innovative top-down physical method, which is free of chemical waste that could directly harm the environment. The nanoparticles were first characterized by UV/vis, DLS, Zeta potential, TEM and SAXS, and biological tests were further performed to evaluate cytotoxicity and cell death metabolism. Characterization of AuNPs revealed an absorption peak at 537 nm, with moderate stability according to the ζ potential. Transmission electron microscopy evidenced a higher frequency of spherical nanoparticles with diameters around 15–20 nm. These results were consistent to the ones presented by SAXS, in which a polymeric outer core–shell was seen in spherical AuNPs. The biological results demonstrated biocompatibility of AuNP, presenting no cytotoxic activity after 24–48 h of exposure at up to 20 μg/mL against MRC-5 cells. Gold nanoparticles were able to induce initial apoptosis only at higher concentrations (>35 μg/mL), possibly through the accumulation of AuNPs in autophagosomes, as observed in indirect immunofluorescence tests. This work was effective in producing biocompatible AuNPs, using the magnetron sputtering technique on powdered solid substrates, standing out as a clean, fast, and low-cost method. Additionally, AuNPs can be potentially applied at minimal concentrations, boosting the use in several biotechnology and health applications.

## Graphical abstract

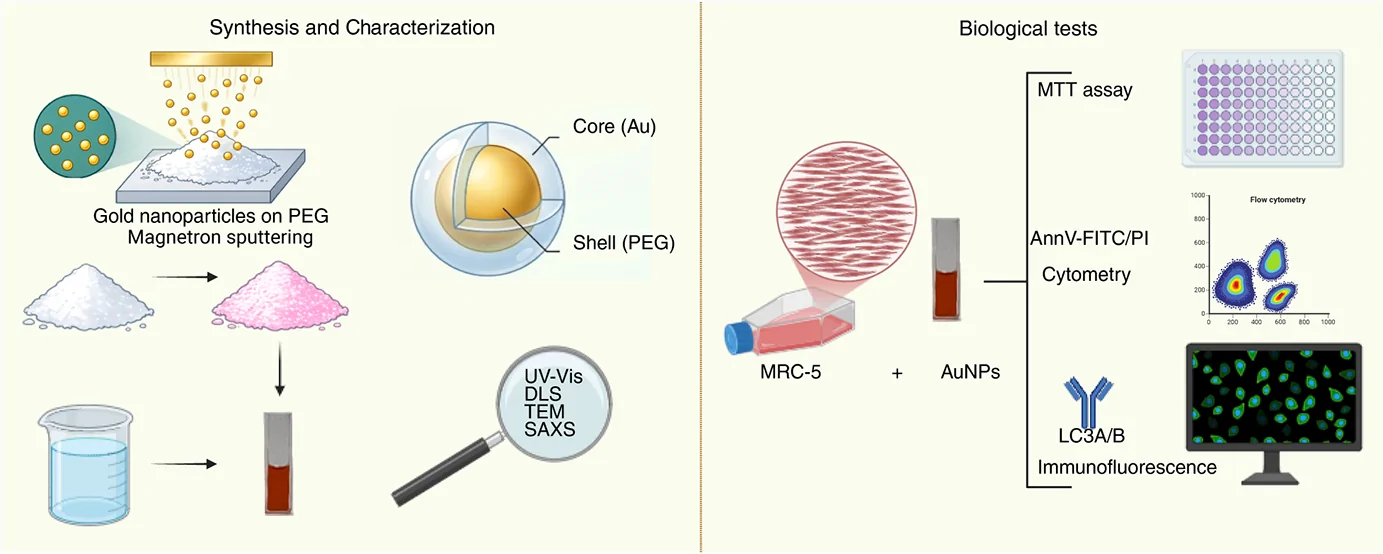

## Introduction

The field of gold nanoparticles (AuNPs) for medical applications has rapidly expanded with the advancement of new technologies. The effort to understand and mitigate the toxicological risks associated with AuNPs reinforces attention to the concept of green toxicology, which contributes to public health safety and environmental preservation.

AuNP synthesis can occur through chemical (bottom-up) or physical (top-down) methods. Yet, chemical approaches have limitations, including the biocompatibility of NPs and their environmental concerns. Therefore, green synthesis methods have been developed to reduce the use of toxic reagents. However, nanoparticle chemical synthesis can take days and still involves reagents such as alcohols and acids.^1–5^ Traditional physical synthesis using ionic liquids also has limitations, as nanoparticles are trapped in high-value-added liquids, which can pose biocompatibility and environmental risks. New techniques are constantly being developed to overcome the challenges in nanoparticle synthesis, as industries are interested in these materials because of their unique properties and broad applications.^6,7^ In this sense, physical synthesis through magnetron sputtering on solid phase has been explored by our research group and demonstrated promising results, reducing environment and health impacts.^8^

In general, gold nanoparticles have low toxicity compared with other metals and are presented in many sizes and shapes. They can be processed individually or conjugated with drugs, antibodies, peptides, and DNA.^3,9^ Besides being used as delivery vehicles, a variety of applications are available because of their surface plasmon resonance (SPR) characteristic, which allows them to absorb and emit heat at a specific wavelength. The development of biocompatible nanoplatforms for therapeutic delivery remains a major focus in nanomedicine; for instance, Wang et al. recently engineered a tea polyphenol-based nanocarrier that demonstrated excellent biocompatibility and efficient cytosolic protein delivery, highlighting the translational potential of well-tolerated nanomaterials.^10^

This feature can be utilized for photothermal therapy, laser ablation, imaging, and biosensors, among other applications.^11,12^ Beyond gold-based systems, other inorganic nanocarriers such as copper carbonate nanoparticles have also been developed as effective biomineralized platforms for macromolecular drug delivery and multimodal therapy, underscoring the growing interest in safe and multifunctional nanomedicines.^13^ Research has been conducted to assess the cytotoxicity of various AuNPs, but literature is controversial since it is challenging to compare different biological tests and methods of production, purification, and functionalization.^14^ The importance of rigorous biocompatibility assessment is further reinforced by recent studies on advanced biomaterials; for example, Yang et al. demonstrated that modified tilapia skin matrices maintained excellent biocompatibility, underscoring the critical role of ISO 10993-based evaluations in the development of new biomedical constructs.^15^ Green engineering aims to incorporate toxicological considerations into the early stages of nanoparticle development and minimize adverse health and environmental impacts without compromising the functional benefits of the AuNPs.

In this study, we synthesize gold nanoparticles using an innovative, fast, and cost-effective method through a magnetron sputtering process on a solid phase. AuNPs were first characterized through physical-chemical parameters and tested against human fibroblast cells, in which cytotoxicity effects and cell death patterns were evaluated as a function of concentration.

## Results and Discussion

### Synthesis and Characterization of Gold Nanoparticles

In this study, we present a new technique developed by our research group in which gold nanoparticles were synthesized on a polyethylene glycol powder substrate using the magnetron sputtering technique^8^ A color change in the polymer from white to dark pink was observed, consistent with the localized surface plasmon resonance characteristic of AuNPs. After solubilization, the AuNP colloidal solution was analyzed by UV–Vis spectrophotometry, revealing an absorption peak at 537 nm, which is typical of nanoparticles of this metal (Figure 1).

**Figure 1:**
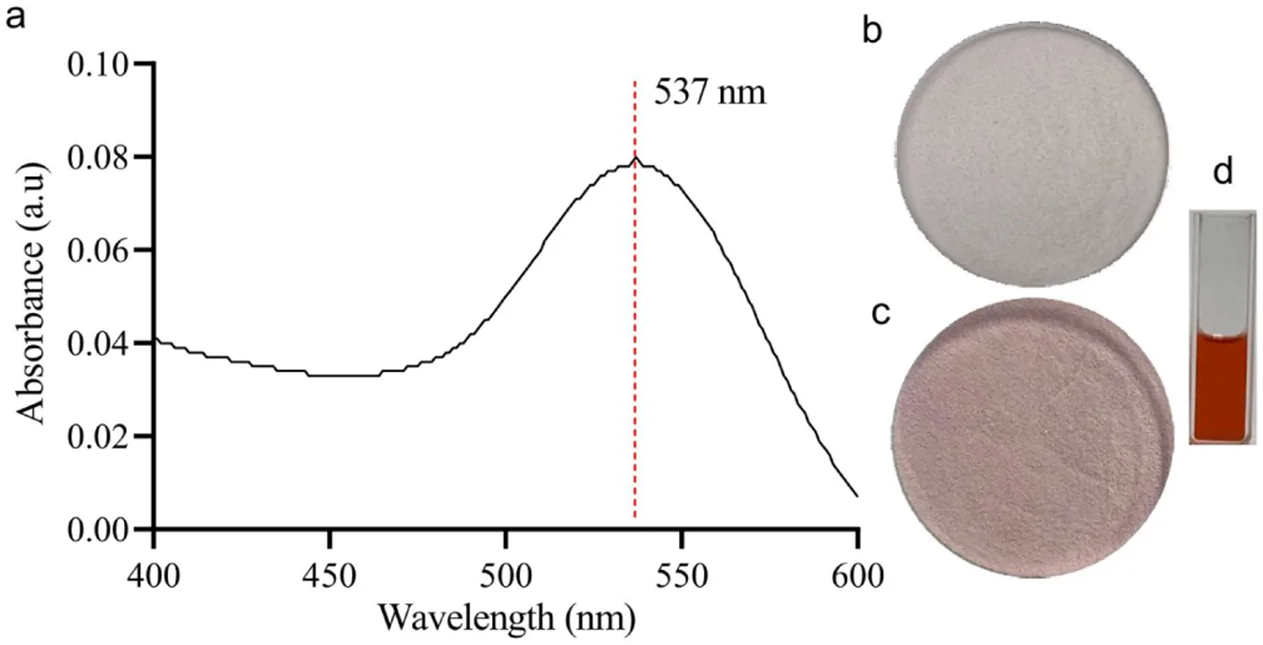
UV–vis spectrum of the colloidal solution of gold nanoparticles, revealing an absorption peak at 537 nm; a) UV–vis–UV–vis; b) PEG before sputtering; c) PEG@AuNPs later sputtering; d) colloidal solution after dilution of PEG@AuNPs.

The standardization of gold nanoparticle synthesis is complex and requires the careful control of several parameters, such as deposition energy, liquid type, target distance, and other factors that can significantly influence the process and result in nanoparticles with diverse characteristics (Table 1).^16^ In general, polyethylene glycol is widely used in the liquid-phase synthesis of nanoparticles, serving both as an ionic liquid and as a coating agent to enhance the stability and improve interactions in biological systems. However, its use in a solid granular form has rarely been reported, representing a promising alternative approach with potential applications and an enhanced stability. Our research group has published recently a paper using AuNPs synthesized by this method with application in immunoassays.^8^

**Table 1:** Recent Reports on the Magnetron Sputtering Technique for Synthesizing AuNPs in Polyethylene Glycol^a^

Author	Current (mA)	Distance (mm)	Argon (Pa)	Time (min)	Substrate (g/mol)	TEM (nm)	DLS (nm)	Zeta (mV)
Pislova et al., 2021 ^17^	30	50	8	5	PEG 600	6,4	14.9 ± 3.4	–16.4 ± 0.8
					PEG + Chitosan	6	15.3 ± 3.0	867.1 ± 35.5
					PEG + MethylCellulose	7,8	8.1 ± 1.2	23.8 ± 11.2
Reznickova et al., 2019 ^18^	30	50	10	5	PEG	5.6 ± 1.9	9.2 ± 1.5	NS
					PEG-SH	1.5 ± 0.3	3.1 ± 0.7	NS
					PEG-NH2	2.5 ± 0.8	4.3 ± 0.9	NS
				15	PEG 400	5.9 ± 1.9	8.8 ± 2.2	–24.7 ± 4.5
					PEG-SH	1.6 ± 0.3	5.4 ± 0.6	–29.2 ± 1.6
					PEG-NH2	2.9 ± 0.8	4.4 ± 1.1	–47,0 ± 1,3
Hatakeyama et al., 2011 ^19^	20	25	16–19	50	PEG 600	2–8 nm	NS	NS
Nguyenova et al., 2024 ^20^	30	50	10	15	PEG	3.8 ± 0.3	NS	–19.7 ± 2.8
					PEG-NH2	3.8 ± 0.1		–2.1 ± 0.7
Sumi et al., 2015 ^21^	10	25	2	10	PEG 600	4.8 ± 0.8	NS	NS
				20		5.2 ± 1.3		
				30		5.5 ± 1.5		
				40		4.4 ± 0.4		
	20			10		5.4 ± 1.1		
				20		5.3 ± 1.1		
				30		5.0 ± 0.6		
				40		5.0 ± 0.5		
	30			10		5.1 ± 2.8		
				20		5.4 ± 0.8		
				30		5.6 ± 1.2		
				40		4.7 ± 0.6		

a NS: Not specified.

The DLS technique is based on the detection of the intensity of light scattered by particles in suspension, providing information about the average particle size and the size distribution of the nanoparticles.^22^ At the same time, the ζ potential can also be measured, providing information about the electrostatic repulsion between particles with similar charges and its correlation with their tendency to aggregate. ζ potential values above −10 mV generally indicates unstable particles with a high probability of aggregation, whereas values between −10 and −30 mV indicate moderate stability. In contrast, values greater than −30 mV are associated with high colloidal stability due to strong electrostatic repulsive forces.^22–24^

The colloidal solution in water exhibited a net ζ potential of −10.5 mV (with an experimental variation of approximately ±5 mV) and a hydrodynamic diameter of 152.8 nm. Although a surface charge magnitude around 10 mV typically suggests weak electrostatic repulsion under classical DLVO boundaries, the colloid displayed long-term stability. This behavior is reconciled by the fact that steric hindrance, rather than pure electrostatic repulsion, governs the colloidal stability of these systems. The highly hydrophilic PEG chains grafted onto the AuNPs swell substantially in aqueous media, creating a physical steric barrier that prevents core–core contact and subsequent aggregation. Furthermore, the presence of this thick, neutral polymer shell physically shifts the shear plane further away from the inorganic gold core, which naturally attenuates the measured ζ potential at the boundary. In contrast, when the AuNPs were diluted in Dulbecco’s Modified Eagle Medium (DMEM) for biological assays, the nanoparticles exhibited a ζ potential of −16 mV and an increased hydrodynamic diameter of 457 nm (Figure 2b,c). This transition is attributed to the adsorption of culture medium proteins, forming a protective protein corona that enhances electrosteric stabilization, a phenomenon widely documented for polymer-coated nanoparticles in biological fluids.^22,24^

**Figure 2:**
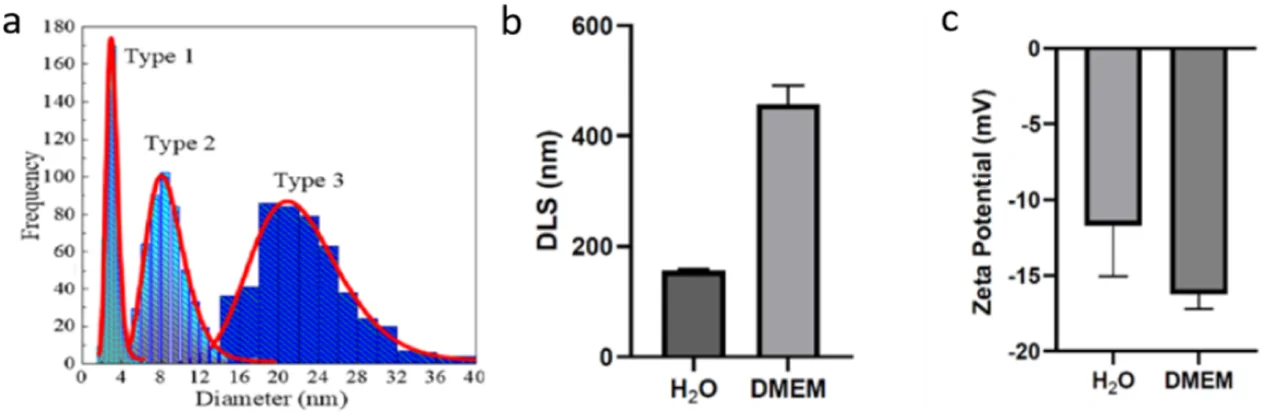
Frequency distribution of nanoparticle diameters by TEM (a) DLS analysis (b) and zeta (ζ) potential (c).

Transmission electron microscopy revealed polydisperse nanoparticles with various shapes, predominantly spherical AuNPs with three types of diameters (Figure 2a, Figure 3). The distribution evidenced a higher frequency of nanoparticles with diameters of around 15–20 nm. Two other groups of sizes were highlighted with average sizes of 4 and 8 nm. The presence of nanoparticles with distinct size distributions further reflects the nanoparticle formation processes. To confirm that all nanoparticles and clusters were of gold composition, EDS images were taken (Figure 3c,d).

**Figure 3:**
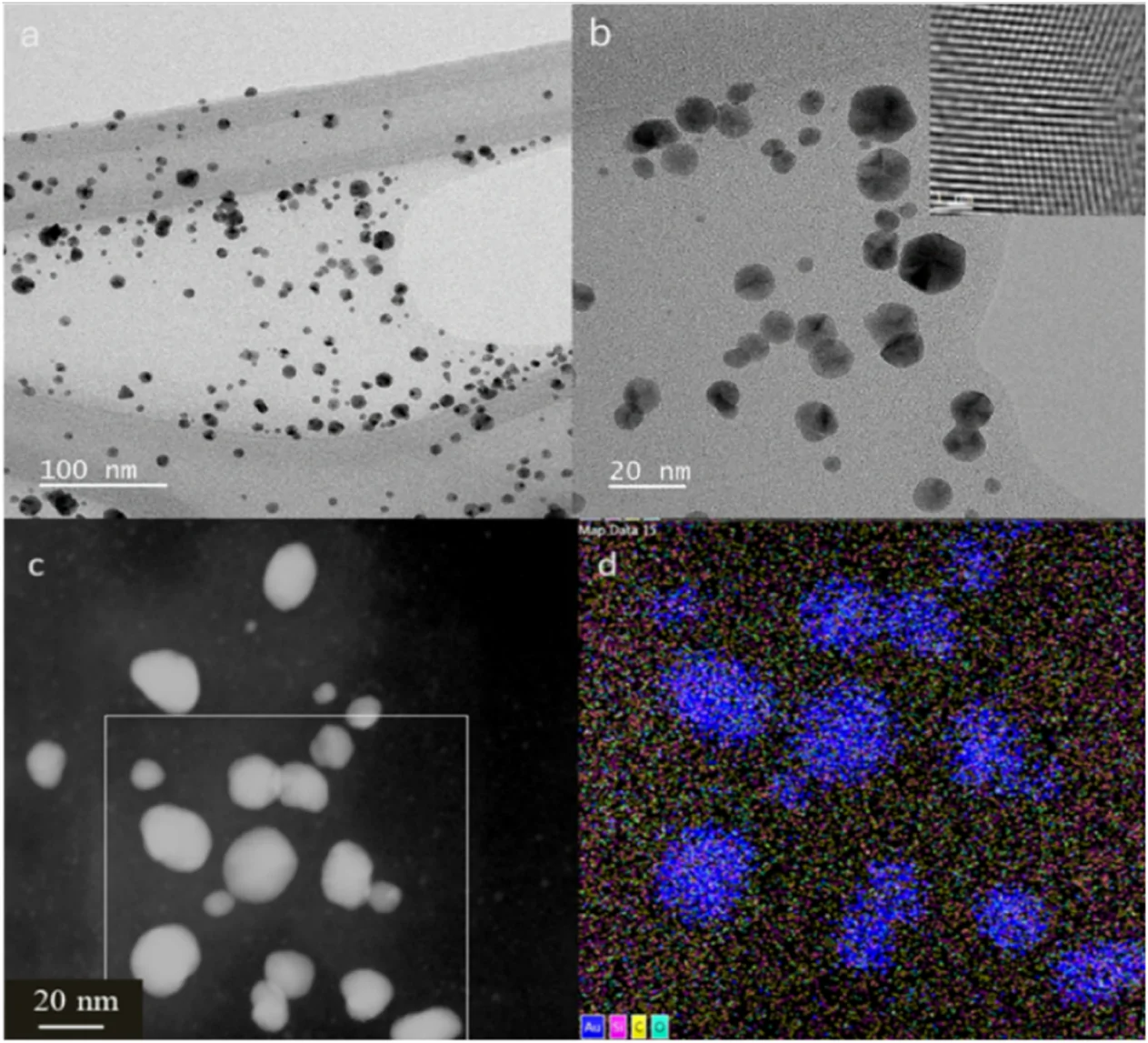
Transmission electron microscopy of AuNPs (a, b) and energy-dispersive spectroscopy (EDS) assay for elemental identification (c, d).

This result highlights discrepancies between the DLS and TEM analyses, likely arising from the strong affinity of gold for organic molecules and from the inherent differences between the analytical techniques. While DLS measures the effective hydrodynamic diameter of hydrated, swollen PEG-grafted clusters in aqueous suspension, TEM requires sample deposition and drying under high vacuum. Upon dehydration, the flexible, water-swollen polymer chains undergo physical collapse onto the gold core into a condensed layer. Furthermore, due to the lack of heavy-metal staining, bright-field TEM imaging is dominated by electron scattering from the high-density gold core, rendering the low-contrast, collapsed organic shell virtually invisible. Further investigations were performed to evaluate the core–shell architecture of these nanoparticles, as well as the presence and integrity of the polyethylene glycol coating layer through small-angle X-ray scattering analysis.

### Small-Angle X-ray Scattering

SAXS analysis was conducted to evaluate the core–shell structure among the AuNP and polyethylene glycol, leading to the formation of what is hereafter termed Au@PEG, or PEG-coated gold nanoparticles. The samples were prepared using both Milli-Q water and 70% ethanol as solvents, since the scattering length densities of water and PEG are similar, which can lead to some model fitting issues such as high standard errors and unprecise parametrization. To circumvent this issue, 70% ethanol was used as a solvent, though not representative of application conditions, it proved efficient in providing higher contrast for the PEG shell and served as a good comparison basis for the water-based model.

The SAXS curves for water and ethanol-solvated Au@PEG are seen in Figure 4, wherein the fitted models are superimposed, and their respective residuals are shown. Table 2 reports the details and results of the fitting procedures, where both samples show the presence of low-q structures, probably in the form of agglomerates. Converting the gyration radius (Rg) into a spherical radius (R = 1.29 × Rg), mean particle sizes of 334.52 and 369.92 nm were obtained for water and ethanol Au@PEG samples, respectively. An Rg growth occurred for the ethanol-based Au@PEG, possibly a consequence of the poorer solvent causing more cluster aggregation due to relaxation of the PEG shell. Also, an apparent discrepancy with the hydrodynamic diameter of 152.8 nm obtained via DLS in water can be attributed to the different physical weighting of the two techniques. Because small-angle X-ray scattering intensity scales with the square of the particle volume and SLD (∝ (Δ*ρ*)^2^, *V* ^2^), the SAXS profile at low angles is exceptionally sensitive to and heavily dominated by trace amounts of larger aggregates composed of gold cores. Conversely, dynamic light scattering (DLS) is highly responsive to the diffusion of the dominant, stable population of smaller oligomeric core–shell clusters (∼153 nm) suspended in the aqueous medium. The difference from the water-based Au@PEG agglomerate radius to the DLS hydrodynamic diameter of 457 nm is itself indicative for the presence of solvated-PEG shell around agglomerates.

**Figure 4:**
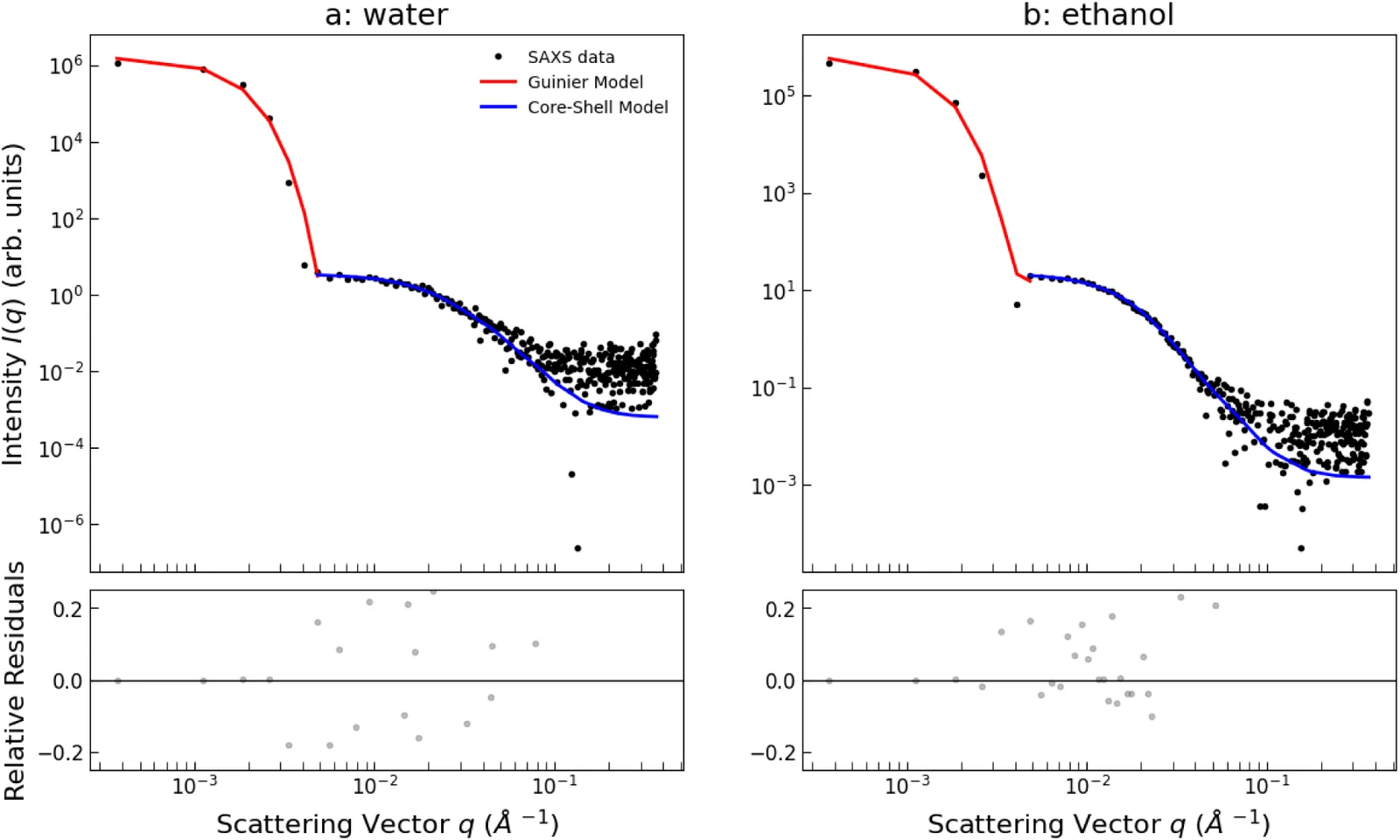
SAXS results for a) Au@PEG in water and b) Au@PEG in 70% ethanol. Plots show Guinier (red) and core–shell (blue) fits, as well as the relative residuals of the plots.

**Table 2:** Fitting Details and Results for Au@PEG Samples in Water and 70% Ethanol

Guinier Fit Details			
Sample	Scale (a.u)	Background (a.u)	Rg (nm)
Au@PEG in Water	1.62 × 10^6^ ± 2.13 × 10^3^	2.97 × 10^–3^ ± 7.09 × 10^–4^	129.66 ± 0.04
Au@PEG in 70% Ethanol	6.37 × 10^5^ ± 1.96 × 10^3^	4.84 × 10^–3^ ± 7.15 × 10^–4^	143.38 ± 0.16

Spherical Core–Shell Fit Details						
Sample	Scale (a.u)	Background (a.u)	Core Radius (nm)	Shell Thickness (nm)	Radius Distribution (log-normal)	Thickness Distribution (Gaussian)
Au@PEG in Water	5.88 × 10^–5^ (fixed)	7.34 × 10^–4^ (fixed)	3.72 ± 1.48	15.66 ± 5.77	0.44 ± 0.18	0.00 ± 0.00 (fixed)
Au@PEG in 70% Ethanol	1.54 × 10^–5^ (fixed)	1.39 × 10^–4^ (fixed)	6.59 ± 0.62	9.06 ± 2.39	0.39 ± 0.05	0.00 ± 0.00 (fixed)

Moving beyond clustering phenomena, the core–shell models successfully confirmed the presence of a PEG outer shell among primary particles in the size distribution. Unlike the Guinier model, in which no geometric information is assumed *a priori*, the core–shell model typically assumes a spherical form factor. It can be seen from the model that the *qr* specific terms are factored by contrast terms, which are defined by the core, shell, and solvent SLDs. Therefore, if converged, the model can successfully resolve the parameters to each component of the core–shell structure, which is what was observed for both Au@PEG samples.

Table 2 reports core radius of 3.72 ± 1.48 and 6.59 ± 0.62 nm and shell thicknesses of 15.66 ± 5.77 and 9.06 ± 2.39 nm for the water and ethanol-based Au@PEG dispersions, respectively. While PD values (0.44 and 0.39 for water and ethanol-based samples, respectively) for core sizes seem to match the distributions observed by TEM, the shell PD was fixed as 0.00 due to the necessity of lowering calculation instabilities. As such, the model successfully resolved a homogeneous effective mean thickness layer for the polymer coating, as observed. Consequently, the high standard deviation in the case of the aqueous Au@PEG can be ascribed both to the lack of factorizing a distribution function D­(R) to the resolution of the form factor P­(q) and the lower electron density contrast between PEG and water. Despite these limitations, marked differences in both core radii and shell thicknesses are evident. The smaller core radius observed in water suggests a highly efficient and rapid stabilization process driven by solvent quality. Upon dispersion, the high dielectric constant of water provides immediate electrostatic repulsion, while concurrently promoting an abrupt swelling of the PEG chains into an extended conformation, as evidenced by the thicker 15.66 nm shell. This highly solvated polymeric shell acts as the primary stabilizing agent via steric repulsion, compensating for the low electrostatic charge of the particles and ensuring colloidal integrity even under low zeta potential conditions. This steric hindrance traps and stabilizes the primary gold clusters at a smaller dimension. In contrast, 70% ethanol acts as a poorer solvent with a lower dielectric constant. This environment compresses the electrostatic repulsion and leads to a relatively contracted PEG shell (reflected by the thinner 9.06 nm measurement). This temporary reduction in colloidal protection may facilitate primary nanoparticles’ partial coalescence before the polymer fully encapsulates them, ultimately yielding the larger effective core size detected by SAXS.

Nanoparticles of three main shapes were observed, although spherical morphology was predominant. Furthermore, the AuNPs exhibited a trimodal diameter distribution (Figures 2a, 3), which is consistent with the SAXS observations of core size variations as a function of solvent quality. The most frequent nanoparticle sizes corresponded to type III, ranging from 15 to 20 nm. Two other size populations were observed, namely, Type I and Type II, with average sizes of approximately 4 and 8 nm, respectively. Notably, types II and III closely match the primary core sizes derived from the water- and ethanol-based Au@PEG core–shell models. From these results, it can be concluded that the smaller Type I and II particles (Figures 2a, 3) may coalesce to form the larger Type III AuNPs (Figure 5). These larger particles exhibit significant polydispersity and possess more than two crystalline domains (Figures 2a, 3). In contrast, the AuNPs of type I and II particles are smaller and have fewer domains. The presence of crystalline planes with multiple orientations indicates that particle coalescence was strongly favored, resulting in a higher prevalence of type III nanoparticles.^25–27^

**Figure 5:**
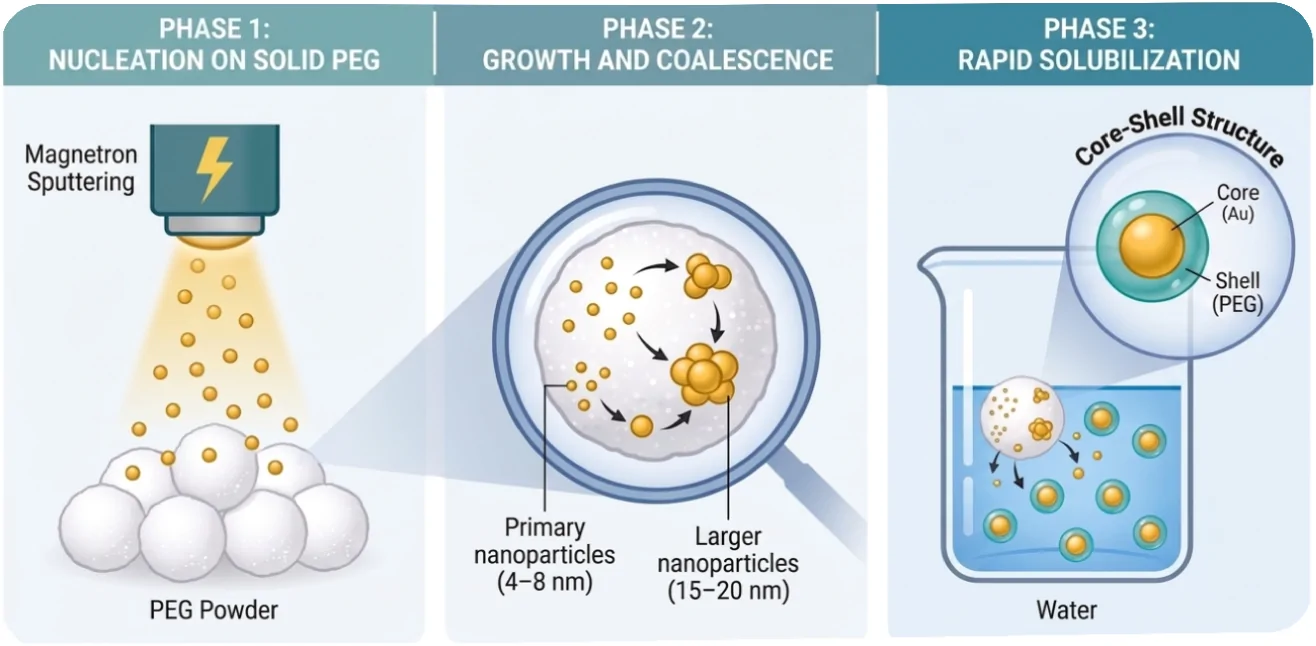
Schematic illustration of the proposed mechanism governing the formation of AuNP on PEG substrate using magnetron sputtering. Phase 1 represents the nucleation of AuNP on solid PEG, followed by a phase of growth and coalescence, which guaranties a rapid solubilization of the core–shell formed by Au and PEG. This figure was generated using NotebookLM and reviewed for scientific accuracy by the authors.

A schematic illustration of the proposed mechanism governing the formation of AuNP on PEG substrate using magnetron sputtering is illustrated in Figure 5.

### *In Vitro* Analysis of Cell Viability and Morphology After AuNP Exposure

Once AuNP enter inside the cell, these nanostructures can accumulate in the cytoplasm or in organelles known as autophagosomes.^28^ Understanding mechanisms of cytotoxicity or excretion is crucial to determine an efficient application for each condition tested. A biocompatible profile of concentrations up to 20 μg/mL (76.46 ± 3.09) of AuNP were observed, with cell viability >70% (Figure 6). The results are in accordance with ISO 10993-5:2009,^29^ *in vitro* cytotoxicity tests with results below 30% should be considered noncytotoxic material. That means they do not produce toxic effects sufficient to compromise the integrity of a cellular or biological system. Generally, nanomaterials retaining >70% cell viability are also considered safe for use in biological applications.^29,30^ The lowest concentration (5 μg/mL) showed >90% of cell viability, a value three times below the threshold for cytotoxicity according to ISO 10993-5:2009 regulations.^29^

**Figure 6:**
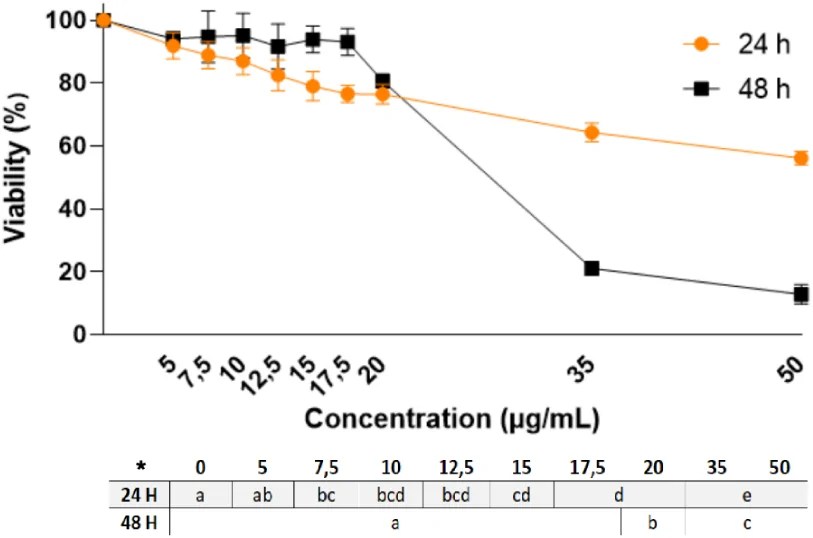
MTT assay in human lung fibroblast cells (MRC-5) treated with gold nanoparticles for 24 and 48 h. *One-way ANOVA test with Tukey’s correction (*p* < 0.05).

Longer exposure (48 h) of 20 μg/mL (80.62 ± 2.25) AuNP maintained cell viability, with an increase in cytotoxicity only at concentrations of 35 and 50 μg/mL (21.04 ± 2.15 and 12.78 ± 3.12 respectively) (Figure 6). However, concentrations of 20 μg/mL and lower surpassed the cytotoxicity caused in the first 24 h and showed cell recovery, with cell proliferation close to 100%, an excellent perspective for cell metabolism. Cells may return to their normal function after exposure to nanoparticles if their machinery remains intact. However, if exposure occurs at high concentrations sufficient to cause several molecular interferences on the cells, and the NPs are easily taken up by the cell accumulating in its interior, the cells tend not to recover, leading to cell death and population decline.^31,32^

The uptake of nanomaterials by cells is associated with the intrinsic characteristics of the nanoparticles.^33^ Size, shape, and surface charge directly influence the degree of cytotoxicity. Nanoparticles smaller than 5 nm can cause more deleterious effects, as they can penetrate various organelles, leading to toxic effects associated with ROS generation, while particles larger than 20 nm tend to produce fewer harmful effects.^34^

Due to the complex nature of nanomaterials, conflicting studies have led to different viewpoints regarding their safety. It is clear that establishing a standard biosafety label for AuNPs is challenging given the various parameters involved. In fact, the biocompatibility of AuNPs is strongly influenced by the characteristics of the nanoparticles, the biological target, and the methodology used to assess their cytotoxicity.^35^ We observed that cells treated with 35 and 50 μg/mL exhibited changes such apoptotic bodies and an accumulation of dark spots in the cytoplasm, indicating the presence of these nanostructures (Figure 7).

**Figure 7:**
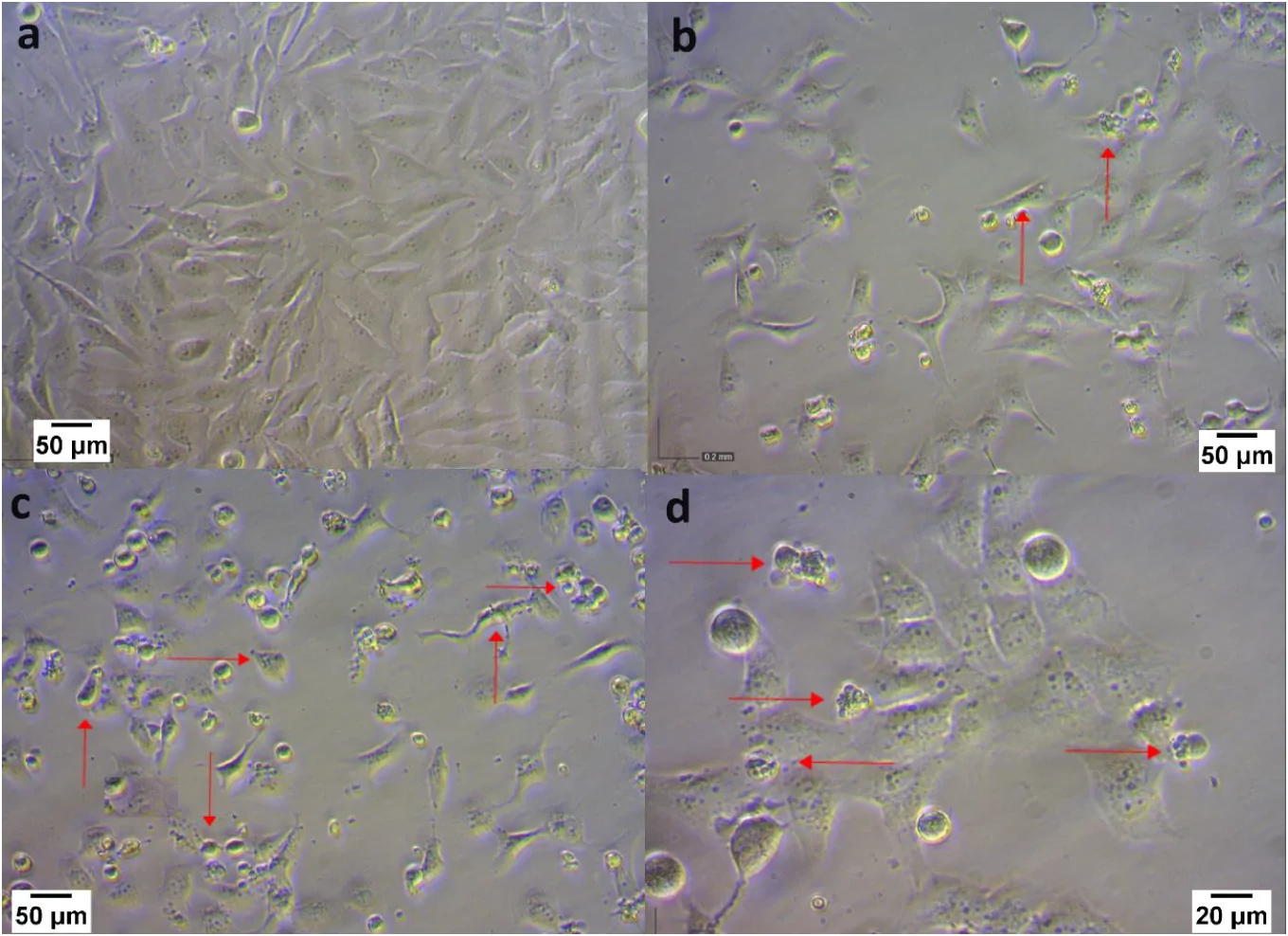
MRC-5 cell line treated with AuNPs for 24 h and their aspect regarding changes in morphology; a) untreated cells (200×); b) 35 μg/mL (200×); c) 50 μg/mL (200×); d) 50 μg/mL (400×).

Mechanical effects may occur due to the intracellular accumulation of AuNPs, and these effects are related to the dosage delivered to the cells. Several authors report the use of very low concentrations for *in vivo* applications, without need for high concentrations to achieve the desired results.^36^ The use of AuNPs up to 20 μg/mL proved to be feasible and safe regarding cytotoxicity parameters for fibroblast cells (MRC-5), as demonstrated in the cell viability assay. Higher concentrations may lead to accumulation in the cytoplasm, resulting in effects such as apoptosis, which can be indicated by changes in cellular morphology.^37,38^

### *In Vitro* Analysis of AuNP Internalization in Cells and the Cell Death Response

Cell death parameter after AuNP exposure by flow cytometry using the Annexin V-FITC/PI Apoptosis Kit (Sigma-Aldrich, MO, USA) was first analyzed. The negative control is represented by cells without AuNPs (Figure 8) and this group remained viable after 24 h. Treatments with 35 and 50 μg/mL of AuNPs induced apoptosis rates of 61.2 ± 8.48 and 62.5 ± 6.08% in cells, respectively (Figure 8). Note that both 35 and 50 μg/mL of AuNP generated similar cell death profiles by flow cytometry, indicating early apoptosis events, which may be potentially reversible when compared to late apoptosis. Apoptosis is the main mechanism of cell death and is associated with deleterious changes in the cell, with the main signs represented by membrane blebbing, chromatin condensation, cell fragmentation, and morphological changes in the cytoskeleton.^36^

**Figure 8:**
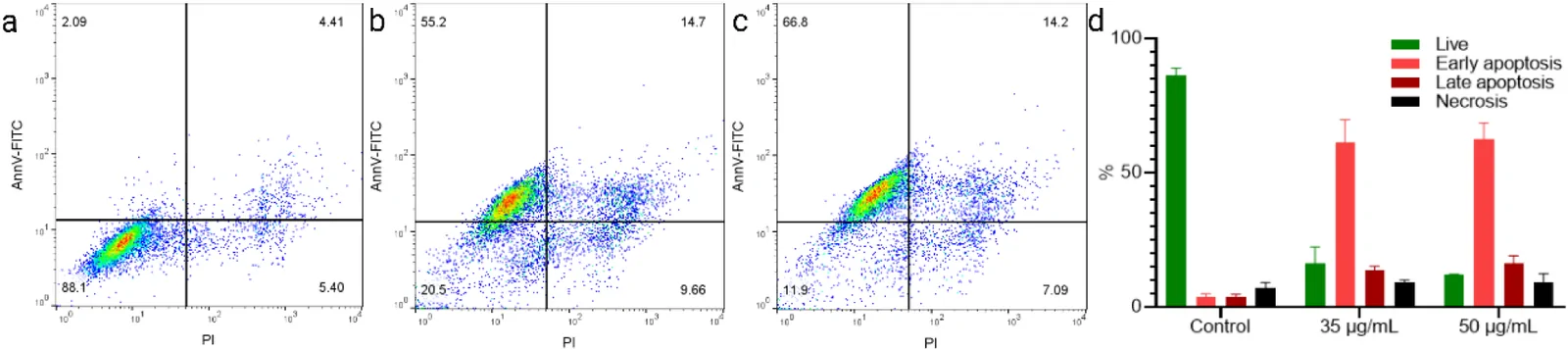
Flow cytometry with annexin/PI to assess the viability of MRC-5 cells; a) negative control; b) cells treated with 35 μg/mL; c) cells treated with 50 μg/mL; d) mean and SD of control, 35 μg/mL and 50 μg/mL.

Events related to early apoptosis may indicate an increase in the production of reactive oxygen species (ROS) and other metabolic changes such as the autophagy regulation pathway (LC3 I and II).^39,40^ The literature points out that treatment with AuNPs can lead to ROS generation and may be involved in the initiation of apoptosis; however, this effect seems to be less pronounced and different from what is observed with bare nanoparticles or those coated with molecules other than PEG.

In order to understand part of the mechanism involved in AuNP cell internalization, the levels of expression of the LC3 A/B protein associated with autophagic processes were investigated. One theory is that the autophagic pathway is used by the cell to maintain cellular homeostasis, storing or capturing all exogenous material, such as AuNPs, as well as damaged or defective organelles and intracellular materials. Autophagosomes act as barriers by accumulating exogenous and harmful material and also help to degrade damaged organelles. The accumulation of autophagosomes may indicate a high concentration of AuNPs in the cytoplasm, as well as being a mechanism to maintain cellular homeostasis.^41,42^

Immunofluorescence assay evaluating levels of expression of the LC3 A/B protein was performed and provided evidence of AuNP accumulation in cells at concentrations above 20 μg/mL (Figure 9). Green fluorescence highlights the expression of the LC3 A/B protein inside the autophagosome once AuNP concentration increases. A comparative aspect can be observed with the group of cells treated with rapamycin, a positive control molecule that induces LC3 A/B expression (Figure 9 – second line). Cells exposed to 35 μg/mL of AuNPs presented higher LC3 A/B expression (green fluorescence) compared to cells exposed to 20 μg/mL of AuNPs. In contrast, cells exposed to lower concentrations (20 μg/mL) of AuNPs showed little or no morphological changes, confirming the hypothesis that cytotoxicity may be related to cellular uptake/accumulation at higher concentrations (>35 μg/mL) of AuNPs and their consequent damage. Lower AuNP concentrations could preserve cell morphology, without apparent compromise of the cell membrane. At the concentration of 20 μg/mL, LC3 A/B autophagosome protein expression was observed in few cells, but this expression intensified at a concentration above 35 μg/mL (Figure 9). The autophagosomes seem to be clustered inside the cytoplasm and indicate that treatment with AuNPs induces their formation, possibly also associated with oxidative stress and AuNP accumulation in the cytoplasm in a dose-dependent manner, acting to protect the cell from succumbing to cellular stress.^41,43–45^

**Figure 9:**
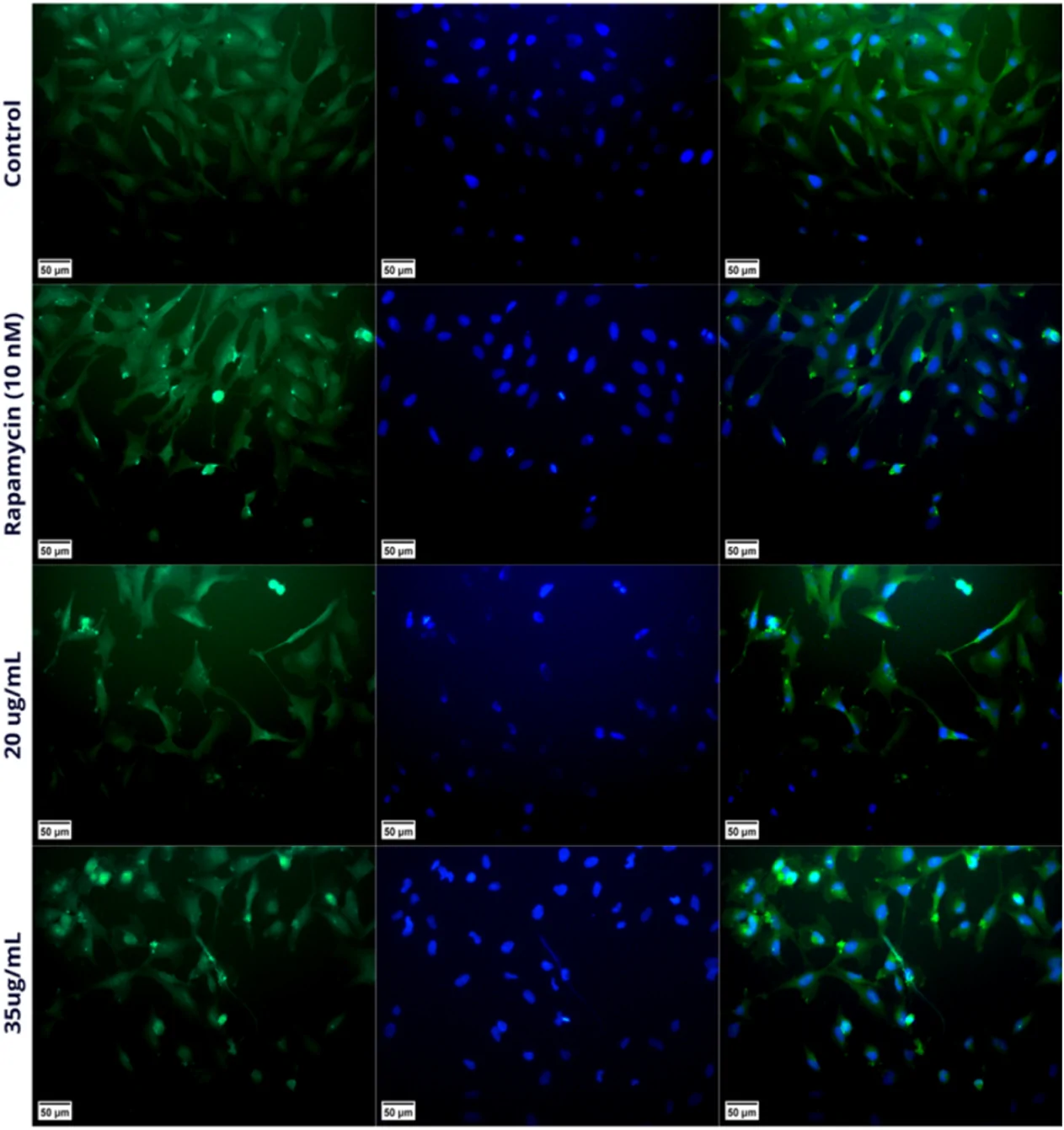
Fluorescence microscopy revealing the expression of the LC3 A/B protein (green fluorescence) for MRC-5 cells treated with 20 and 35 μg/mL; scale bar 50 μm.

The formation of these structures seems to be related to the cell’s capacity to maintain balance while dealing with oxidative stress but also mechanical overload. It seems that parameters such as coating agent must be carefully evaluated to understand the cause-effect relationship that AuNPs may produce in response to dosage and accumulation.^43,46^

The use of gold nanoparticles at low concentrations has been reported as beneficial for balancing cellular homeostasis in stem cells with active inflammatory activity as their internalization triggers the formation of autophagosomes. The activation of this system can increase autophagosome-lysosome fusion and lysosomal degradation, restoring cellular homeostasis and serving as a target model for future therapies in different cell lines.^44^ This hypothesis is consistent with the results here presented, where cell viability was restored after 48 h, showing less than 20% cytotoxicity, suggesting that their use could be safe and effective for several applications, as long as no morphological and irreversible cell death effects are observed in cells.

As previously mentioned, the effect of nanoparticles is attributed to their intrinsic properties. These factors can be enhanced by the administered dose. From these results, we can suggest that apoptosis induction occurs after the cell attempts to maintain balance in the face of various stresses caused by the massive presence of AuNPs in the intracellular environment. The formation of autophagosomes is one of the possible mechanisms that can be recruited to maintain cell integrity and indicates that cellular uptake of gold nanoparticles at low concentrations was directed to the organelle to be stored and digested. At high concentrations, it is most likely that cellular signaling will be destabilized and cellular homeostasis disrupted, potentially making it an alternative for the targeted delivery of products such as drugs or molecules at higher concentrations. The low cytotoxicity of AuNPs exposed to cells, below the cytotoxicity threshold, indicates that normal cells can survive in contact with the nanoparticulate material in an *in vitro* model. The safety profile of gold nanoparticles produced by physical methods as the one used in this study, has been shown to be safe at low doses (>20 μg/mL).

## Conclusions

This study presents results regarding the standardization of gold nanoparticles using the magnetron sputtering technique on powdered solid substrates. This approach stands out as a clean, fast, and low-cost method, especially given the use of powdered polyethylene glycol as a substrate, which offers a long and stable shelf life. The absence of toxic reagents and the simplicity of the process, underscore the success of the objective to study new, more environmentally friendly biotechnological methodologies. In addition, the biocompatibility of gold nanoparticles was evaluated in human fibroblast cell model, showing a dose-dependent profile, with significant cytotoxicity observed only at higher concentrations (>20 μg/mL), and cell recovery observed after 48 h treatment.

Further studies will help standardize and improve this new synthesis method. The nanoparticles can be utilized with a focus on their green toxicology, predicting *in vitro/in vivo* effects based on their physical and chemical properties, and adjusting these parameters during the synthesis process to suit different applications.

## Materials and Methods

### Synthesis of Gold Nanoparticles

The gold nanoparticles were synthesized by physical route by magnetron sputtering in solid substrate on polyethylene glycol (PEG). Briefly, 5 g of PEG were used in a Petri dish for each deposition. The PEG was located at a distance of 20 mm from the Au target (99.99% purity). The equipment was submitted to a base pressure of 5 × 10^–6^ mbar, then argon gas flow was set at 3 sccm. The sputtering time was 10 s. The sample was then removed from the equipment and solubilized with Milli-Q water (1:10 m/v). The AuNPs were stored in a refrigerator at 4 °C at optical density OD 1 (537 nm).

### UV/Vis Spectrum and Dynamic Light Scattering

AuNPs were analyzed by UV–vis spectrophotometer (Thermo Fischer MultiSkan FC) with 400–600 nm to check absorption maximum peak. The hydrodynamic diameter (DLS) and zeta (ζ) potential were evaluated using the Nanosizer instrument (Malvern, Worcestershire, UK). The AuNPs were suspended in ultrapure water, 15 cycles with 15 repetitions were carried out to obtain the average result with the least influence from spurious measurements.

### Transmission Electron Microscopy

For transmission electron microscopy (TEM), samples were pipetted onto copper grids and dried under a vacuum. They were then analyzed using a transmission electron microscope (JEOL 2100F with CCD, EDS, EELS, and precession diffraction) at the Brazilian Center for Research in Physics (CBPF) in Rio de Janeiro/RJ. TEM allowed for the visualization of the gold nanoparticles as well as their shape and size, in addition to quantitative analysis of the average size of the AuNPs and their distribution.

### Small-Angle X-ray Scattering

AuNP suspensions in Milli-Q water and 70% ethanol (1:10 w/v) were analyzed using a Xenocs Nano-inXider, located at the Central Microscopy Laboratory (LCMIC) at the University of Caxias do Sul (UCS). The samples were pippeted into Kapton-windowed cells with a 0.5 mm path length. The equipment operated at the Very High-Resolution preset, at which the copper X-ray tube (λ = 1.5418 Å) operated at 50 kV and 0.6 mA. All runs had an exposure time of 60 s, and the data was collected using a single Pilatus (Dectris) detector module, for which the q interval was 3.7 × 10^–4^ to 0.37 Å^–1^. Data analysis, background subtraction (solvent-filled Kapton cells), scattering length density (SLD) calculations, and 1D scattering pattern model fitting were carried out in SasView software (Table S1).^47^

### Viability Assay

The human lung fibroblasts cell line (MRC-5) was obtained from the American Type Culture Collection (ATCC—CCL-171), Manassas, VA, US. Cells were cultured in DMEM (Dulbecco’s modified eagle’s medium) with high glucose supplemented with 10% fetal bovine serum (FBS) and 1% penicillin-streptomycin. The cell line was maintained in a humidified incubator with 5% CO_2_ at 37 °C. Cells with 80–90% confluency was used for the cytotoxicity assay.

For the cytotoxicity assay, the MTT method (3-[4,5-dimethylthiazol-2-yl]-2,5-diphenyl tetrazolium bromide) was employed. Cells were plated in 96-well plates at a density of 8 x10^4^ cells/mL. After 24 h of incubation, cells were treated with different concentrations (0–50 μg/mL) of gold nanoparticles for 24 and 48 h. DMEM without gold nanoparticles was used as a negative control. Cells were monitored under an inverted microscope (Nikon, TS2-S-SM, Japan), and images were captured using DinoLite capture. MTT solution was added to each well and incubated under the same conditions for 2 h. Dimethyl sulfoxide was added to each well and absorbance was measured using a UV/vis spectrophotometer (Thermo Fischer MultiSkan FC) at 570 nm.^48^ The experiment was conducted in triplicate.

### Evaluation of Cell Death (Apoptosis/Necrosis) through Flow Cytometry

Cells (3 x10^5^ cells/mL) were treated with 35 and 50 μg/mL of gold nanoparticles, with DMEM high-glucose medium used as the negative control. After 24 h treatment, the cells were collected in a test tube and concentrated by centrifugation at 1,500 rpm for 5 min. The supernatant was discarded, and the pellet was resuspended in 300 μL of PBS. Annexin V-FITC/PI Apoptosis Kit (Sigma-Aldrich, MO, USA) was added following the manufacturer’s instructions and incubated for 30 min in the dark, at room temperature. Subsequently, the samples were analyzed using flow cytometry equipment (BD FACScalibur, San Jose, California). The experiment was conducted in duplicate.

### Indirect Immunofluorescence Assay

The MRC-5 line is plated in 6-well plates with 3 × 10^5^ cells/mL. After 24 h of incubation, the cells were treated with AuNPs (35–50 μg/mL) per 24 h in incubator 37 °C and 5% CO_2_. The negative control utilizes DMEM medium and positive control with rapamycin 100 nM. Cells were fixed with glutaraldehyde 2.5% and blocked with 2% bovine serum albumin (BSA) for 30 min. Then primary antibody, monoclonal anti-LC3A/B antibody (product number 4180, Cell Signaling, 1:100 v/v) for 1 h, followed by incubation with secondary antimouse fluorescein isothiocyanate (product number 150077, Abcam, FITC) (Antimouse IgG, Sigma, 1:150 v/v) for 1 h. The images were captured with an optical fluorescence microscope (BX43 – Olympus).^49^

### Statistical Analysis

Statistical analysis was conducted using SPSS 21.0 software. A one-way ANOVA test with Tukey’s correction was performed. The significance level was set at *p* < 0.05.

## Supplementary Material



## Data Availability

Data will be made available on request

## Notes

Models used for the SAXS study of Au@PEG samples in different solvents, transmittances and scattering length densities calculated for the sample constituents (PDF)

## Abbreviations

AuNPs: gold nanoparticles
NPs: nanoparticles
PEG: polyethylene glycol
DLS: dynamic light scattering
TEM: transmission electron microscopy
SAXS: small-angle X-ray scattering
ATCC: American Type Culture Collection
MRC-5: human fetal lung fibroblast
FBS: fetal bovine serum
DMEM: Dulbecco’s Modified Eagle’s Medium
MTT: 3-(4,5-dimethylthiazol-2-yl)-2,5-diphenyltetrazolium bromide
SPR: surface plasmon resonance
SLD: scattering length densities.

## References

[1] Rahmani
Kukia N., Rasmi Y., Abbasi A., Koshoridze N., Shirpoor A., Burjanadze G., Saboory E. (2018). Bio-Effects of TiO2
Nanoparticles on Human Colorectal Cancer and Umbilical Vein Endothelial
Cell Lines. Asian Pac. J. Cancer Prev..

[2] Roy A., Pandit C., Gacem A., Alqahtani M. S., Bilal M., Islam S., Hossain M., Jameel J. M. (2022). Biologically
Derived Gold Nanoparticles and Their Applications. Bioinorg. Chem. Appl..

[3] Dheyab M. A., Aziz A. A., Moradi Khaniabadi P., Jameel M. S., Oladzadabbasabadi N., Mohammed S. A., Abdullah R. S., Mehrdel B. (2022). Monodisperse Gold Nanoparticles:
A Review on Synthesis and Their Application in Modern Medicine. Int. J. Mol. Sci..

[4] Monfared E. H., Fathi-Karkan S., Keshavarzi Z. (2026). Green and Chemical Synthesis of PEGylated
Ginger Gold Nanoparticles for Neuro-Nanomedicine Applications. Sci. Rep..

[5] Chonanant C., Maraming P., Daduang J., Limpaiboon T., Prakrankamanant P., Ngernyuang N. (2025). Green Synthesis of Biocompatible
Gold Nanoparticles Using Andrographis Paniculata Leaves Extract for
Targeted Detection of Human Epidermal Growth Factor Receptor 2-Positive
Breast Cancer Cells. ACS Org. Inorg. Au.

[6] Altammar K. A. (2023). A Review
on Nanoparticles: Characteristics, Synthesis, Applications, and Challenges. Front. Microbiol..

[7] Selmani A., Kovačević D., Bohinc K. (2022). Nanoparticles: From
Synthesis to Applications and Beyond. Adv. Colloid
Interface Sci..

[8] Gomes
Machado K., Felippi de Lima L., de Souza W. V., Checca R., Louzada D. R., Sommer R. L., Streck A. F., Aguzzoli C., Roesch-Ely M. (2025). Environmentally Safe Synthesis of Gold Nanoparticles
for Antigen Detection in CLIA System. ACS Omega.

[9] Mukherjee P., Bhattacharya R., Bone N., Lee Y. K., Patra C., Wang S., Lu L., Secreto C., Banerjee P. C., Yaszemski M. J., Kay N. E., Mukhopadhyay D. (2007). Potential
Therapeutic Application of Gold Nanoparticles in B-Chronic Lymphocytic
Leukemia (BCLL): Enhancing Apoptosis. J. Nanobiotechnol..

[10] Wang H., Tang C., Zhang H., Guo L., Zou C., Zhang S., Liu J., Huang Q., Liu Y., Zhou W., Yang G., Gao X. (2025). A Biocompatible Tea
Polyphenol Nanoplatform for Efficient Cytosolic Delivery of Protein
Therapeutics. J. Controlled Release.

[11] Chuang S.-M., Lee Y.-H., Liang R.-Y., Roam G.-D., Zeng Z.-M., Tu H.-F., Wang S.-K., Chueh P. J. (2013). Extensive Evaluations
of the Cytotoxic Effects of Gold Nanoparticles. Biochim. Biophys. Acta, Gen. Subj..

[12] Wang R., Huang X., Chen X., Zhang Y. (2024). Nanoparticle-Mediated
Immunotherapy in Triple-Negative Breast Cancer. ACS Biomater. Sci. Eng..

[13] Dong L., Ding J., Zhu L., Liu Y., Gao X., Zhou W. (2023). Copper Carbonate Nanoparticles as
an Effective Biomineralized Carrier
to Load Macromolecular Drugs for Multimodal Therapy. Chin. Chem. Lett..

[14] Woźniak A., Malankowska A., Nowaczyk G., Grześkowiak B. F., Tuśnio K., Słomski R., Zaleska-Medynska A., Jurga S. (2017). Size and Shape-Dependent Cytotoxicity Profile of Gold Nanoparticles
for Biomedical Applications. J. Mater. Sci.:
Mater. Med..

[15] Yang Y., Jiang X., Li R., Pan J., Yang B., Zhou X., Huang X., Liu J., Li T., Wu S. (2026). An Innovative Gas Foaming-Assisted Decellularizing Strategy to Construct
Decellularized Tilapia Skin Matrix with Improved Porosity, Piezoelectricity,
and Biocompatibility. Biomater Adv..

[16] Sergievskaya A., Alem H., Konstantinidis S. (2023). Magnetron
Sputtering onto Nonionic
Surfactant for 1-Step Preparation of Metal Nanoparticles without Additional
Chemical Reagents. Nanotechnology.

[17] Pišlová M., Kolářová K., Vokatá B., Brož A., Ulbrich P., Bačáková L., Kolská Z., Švorčík V. (2020). A New Way
to Prepare Gold Nanoparticles by Sputtering – Sterilization,
Stability and Other Properties. Mater. Sci.
Eng..

[18] Reznickova A., Slavikova N., Kolska Z., Kolarova K., Belinova T., Hubalek
Kalbacova M., Cieslar M., Svorcik V. (2019). PEGylated Gold Nanoparticles:
Stability, Cytotoxicity and Antibacterial Activity. Colloids Surf., A.

[19] Hatakeyama Y., Morita T., Takahashi S., Onishi K., Nishikawa K. (2011). Synthesis
of Gold Nanoparticles in Liquid Polyethylene Glycol by Sputter Deposition
and Temperature Effects on Their Size and Shape. J. Phys. Chem. C.

[20] Nguyenova H. Y., Hubalek Kalbacova M., Dendisova M., Sikorova M., Jarolimkova J., Kolska Z., Ulrychova L., Weber J., Reznickova A. (2024). Stability
and Biological Response of PEGylated Gold Nanoparticles. Heliyon.

[21] Sumi T., Motono S., Ishida Y., Shirahata N., Yonezawa T. (2015). Formation and Optical Properties of Fluorescent Gold
Nanoparticles Obtained by Matrix Sputtering Method with Volatile Mercaptan
Molecules in the Vacuum Chamber and Consideration of Their Structures. Langmuir.

[22] Jacquot A. J. P., de Souza W. V., Machado G., Roesch-Ely M., Crespo J. D. S., Bortoluz J., Giovanela M. (2025). Chitosan Composites
Functionalized with Green-Synthesized Silver Nanoparticles from Manacá-Da-Serra
Flowers for the Disinfection of Industrial Wastewater. Processes.

[23] Pourali P., Benada O., Pátek M., Neuhöferová E., Dzmitruk V., Benson V. (2021). Response of Biological Gold Nanoparticles
to Different PH Values: Is It Possible to Prepare Both Negatively
and Positively Charged Nanoparticles?. Appl.
Sci..

[24] Dreaden E. C., Alkilany A. M., Huang X., Murphy C. J., El-Sayed M. A. (2012). The Golden
Age: Gold Nanoparticles for Biomedicine. Chem.
Soc. Rev..

[25] Ostwald W. (1900). Über
Die Vermeintliche Isomerie Des Roten Und Gelben Quecksilberoxyds Und
Die Oberflächenspannung Fester Körper. Z. Phys. Chem..

[26] Wender H., Migowski P., Feil A. F., Teixeira S. R., Dupont J. (2013). Sputtering
Deposition of Nanoparticles onto Liquid Substrates: Recent Advances
and Future Trends. Coord. Chem. Rev..

[27] Thanh N. T. K., Maclean N., Mahiddine S. (2014). Mechanisms
of Nucleation and Growth
of Nanoparticles in Solution. Chem. Rev..

[28] Niżnik Ł., Noga M., Kobylarz D., Frydrych A., Krośniak A., Kapka-Skrzypczak L., Jurowski K. (2024). Gold Nanoparticles (AuNPs)—Toxicity,
Safety and Green Synthesis: A Critical Review. Int. J. Mol. Sci..

[29] International Organization for Standardization. ISO 10993-5:2009(E); ISO, 2009.

[30] Kanz’árová H., Pôbiš P. (2024). The “Big Three” in Biocompatibility Testing
of Medical Devices: Implementation of Alternatives to Animal Experimentation—Are
We There Yet?. Front. Toxicol..

[31] Borgese M., Rossi F., Bonfanti P., Colombo A., Mantecca P., Valdatta L., Bernardini G., Gornati R. (2020). Recovery Ability of
Human Adipose Stem Cells Exposed to Cobalt Nanoparticles: Outcome
of Dissolution. Nanomedicine.

[32] Ibrahim B., Akere T. H., Chakraborty S., Valsami-Jones E., Ali-Boucetta H. (2023). Functionalized Gold Nanoparticles
Suppress the Proliferation
of Human Lung Alveolar Adenocarcinoma Cells by Deubiquitinating Enzymes
Inhibition. ACS Omega.

[33] Xi Z., Zhang R., Kiessling F., Lammers T., Pallares R. M. (2024). Role of
Surface Curvature in Gold Nanostar Properties and Applications. ACS Biomater. Sci. Eng..

[34] Xia Q., Huang J., Feng Q., Chen X., Liu X., Li X., Zhang T., Xiao S., Li H., Zhong Z., Xiao K. (2019). Size- and
Cell Type-Dependent Cellular Uptake, Cytotoxicity and in
Vivo Distribution of Gold Nanoparticles. Int.
J. Nanomed..

[35] Chueh P. J., Liang R.-Y., Lee Y.-H., Zeng Z.-M., Chuang S.-M. (2014). Differential
Cytotoxic Effects of Gold Nanoparticles in Different Mammalian Cell
Lines. J. Hazard. Mater..

[36] Kowsalya E., MosaChristas K., Jaquline C. R. I., Balashanmugam P., Devasena T. (2021). Gold Nanoparticles
Induced Apoptosis via Oxidative
Stress and Mitochondrial Dysfunctions in MCF-7 Breast Cancer Cells. Appl. Organomet. Chem..

[37] Kus-Liśkiewicz M., Fickers P., Ben Tahar I. (2021). Biocompatibility and Cytotoxicity
of Gold Nanoparticles: Recent Advances in Methodologies and Regulations. Int. J. Mol. Sci..

[38] Piktel E., Ościłowska I., Suprewicz Ł., Depciuch J., Marcińczyk N., Chabielska E., Wolak P., Wollny T., Janion M., Parlinska-Wojtan M., Bucki R. (2021). ROS-Mediated Apoptosis and Autophagy in Ovarian Cancer Cells Treated
with Peanut-Shaped Gold Nanoparticles. Int.
J. Nanomed..

[39] Zhang X., Guo X., Kang X., Yang H., Guo W., Guan L., Wu H., Du L. (2020). Surface Functionalization of Pegylated Gold Nanoparticles
with Antioxidants Suppresses Nanoparticle-Induced Oxidative Stress
and Neurotoxicity. Chem. Res. Toxicol..

[40] Ozcicek I., Aysit N., Cakici C., Aydeger A. (2021). The Effects of Surface
Functionality and Size of Gold Nanoparticles on Neuronal Toxicity,
Apoptosis, ROS Production and Cellular/Suborgan Biodistribution. Mater. Sci. Eng..

[41] Sarkar A. K., Kaur G., Seth P., Jana N. R., Jana N. R. (2024). Autophagy-Induced
Nanoparticle-Based Clearing of Toxic Huntingtin Protein Aggregates
from Neuron Cells. ACS Appl. Nano Mater..

[42] Iba T., Helms J., Maier C. L., Ferrer R., Levy J. H. (2024). Autophagy
and Autophagic Cell Death in Sepsis: Friend or Foe?. J. Intensive Care.

[43] Mizushima N., Komatsu M. (2011). Autophagy: Renovation of Cells and
Tissues. Cell.

[44] Yin Y., Tian B.-M., Li X., Yu Y.-C., Deng D.-K., Sun L.-J., Qu H.-L., Wu R.-X., Xu X.-Y., Sun H.-H., An Y., He X.-T., Chen F.-M. (2022). Gold Nanoparticles
Targeting the Autophagy–Lysosome System to Combat the Inflammation-Compromised
Osteogenic Potential of Periodontal Ligament Stem Cells: From Mechanism
to Therapy. Biomaterials.

[45] Li J. J., Hartono D., Ong C.-N., Bay B.-H., Yung L.-Y. L. (2010). Autophagy
and Oxidative Stress Associated with Gold Nanoparticles. Biomaterials.

[46] Li S., Zhang C., Cao W., Ma B., Ma X., Jin S., Zhang J., Wang P. C., Li F., Liang X.-J. (2015). Anchoring
Effects of Surface Chemistry on Gold Nanorods: Modulating Autophagy. J. Mater. Chem. B.

[47] Krzywon, J. ; Adams, M. ; Agouzal, N. ; Alina, G. ; Attala, Z. ; Backman, M. ; Bakker, J. ; Beaucage, P. ; Berger, J. ; Bourne, R. SasView Version 6.1.3.; Zenodo. DOI: 10.5281/zenodo.18352296.

[48] Mosmann T. (1983). Rapid Colorimetric
Assay for Cellular Growth and Survival: Application to Proliferation
and Cytotoxicity Assays. J. Immunol. Methods.

[49] Santos W. F. S., Cantuária A. P.
D. C., Félix D. D. C., Guimarães N. C., de Melo I. C. S. (2024). Complex Patterns
on HEp-2 Indirect Immunofluorescence Assay in a Large Sample Referred
for Anti-Cell Autoantibodies Detection. Front.
Immunol..

