# Supplementary material for: Biocompatibility of Human Fibroblast Cells Exposed to Gold Nanoparticles Synthesized by Environmentally Friendly Route
Source: ACS Omega. 2026 Sep 30;11(40):61517–27. doi: 10.1021/acsomega.6c06962 (PMC13670246; doi:10.1021/acsomega.6c06962)
Supplement: Supplementary file 1 [file ao6c06962_si_001.pdf]

Biocompatibility of human fibroblast cells exposed to gold nanoparticles synthesized by environmentally friendly route

Wellington Vieira de Souza* [a,c], Keilla Gomes Machado [a], Francine Girardello [a], Amanda Poletto Santi [a], Noemi Raquel Checca Huaman [b], Daniel Ramos Louzada [b], Rubem L. Sommer [b], Fernando Joel Scariott [a], Rônei de Almeida Aragão [c], Cesar Aguzzoli [c], Mariana Roesch-Ely* [a,c]

[a] Posgraduate Program in Biotechnology, University of Caxias do Sul, 95070560, Caxias do Sul, Rio Grande do Sul, Brazil.

[b] Brazilian Center for Research in Physics (CBPF), Rio de Janeiro, RJ 22290-180, Brazil.

[c] Posgraduate Program in Materials Science and Engineering, University of Caxias do Sul, 95070560, Caxias do Sul, Rio Grande do Sul, Brazil

* Corresponding author: mrely@ucs.br (Mariana Roesch Ely)

SUPPLEMENTARY INFORMATION

Table S1: Models used for the SAXS study of Au@PEG samples in different solvents, transmittances and scattering length densities calculated for sample constituents.

Models	
Model	Theory
Core-Shell Spheres	$I(q) = scale \cdot$ $\Big\{3\Big[V_c(\rho_c - \rho_s)\frac{\sin(qr_c) - qr_c\cos(qr_c)}{(qr_c)^3} + V(\rho_s - \rho_{solvent})\frac{\sin(qr_s) - qr_s\,co}{(qr_s)^3}$ $+ background$<br>Where<br>V: core + shell volume r_s: core + shell radius<br>V_c: core volume r_c: core radius
Guinier	$I(q) = scale \cdot exp[-q^2 r_g^2/3] + background$<br>Where<br>$r_g$: gyration radius
Scattering Length Densities	
Component	Value (Å $^{-2}$)
MilliQ Water	9.47×10^{-6}
70% Ethanol	7.55×10^{-6}
Au	1.25×10^{-4}
PEG 8000	9.97×10^{-6}
Transmittances	
Sample in Kapton Cell	Value (%)
Au@PEG in H2O	27.0
Au@PEG in Ethanol	33.9
MilliQ Water	31.4
70% Ethanol	36.0
